# Regarding the inference of causality in observational non-controlled studies. Comment on “Assessment of sexual function and its association with quality of life and disease severity in patients with atopic dermatitis”^☆^

**DOI:** 10.1016/j.abd.2026.501448

**Published:** 2026-08-27

**Authors:** Vanessa Rigoni Marcato, Anna Carolina Miola, Paula Rosa Coutinho Goulart Borges Mariottoni, Hélio Amante Miot

**Affiliations:** a Department of Infectology, Dermatology, Imaging Diagnosis and Radiotherapy, Faculty of Medicine, Universidade Estadual Paulista, Botucatu, SP, Brazil; b Trichosis Outpatient Clinic, Faculty of Medicine, Universidade Estadual Paulista, Botucatu, SP, Brazil

Dear Editor,

Chronic diseases such as Atopic Dermatitis (AD), beyond physical symptoms, affect multiple dimensions of life, which include relationships, finances, occupational functioning, psychological well-being, self-esteem, body image, sleep, and affect household dynamics.^1^ We commend Dr. Bressan et al. for addressing the impact of AD on overall sexual function and satisfaction.^2^ Nevertheless, we’d like to highlight some methodological considerations that warrant caution regarding the interpretation of these findings.

Overall quality-of-life measurements vary according to the study population and cultural context.^3^ Although the QS-F/M have been validated in Brazilian samples, their internal consistency should be formally assessed for each study population (e.g., using McDonald’s ω) to ensure measurement reliability.^4^ Additionally, the 10-items of these instruments capture distinct domains of sexuality, which may be differentially affected. The domain-specific information is clinically relevant as it may guide targeted interventions. As the authors did not provide an item-level interpretation, the precision of the information on sexual impairment remains unclear.

The modest sample size results in low statistical power for most comparisons and correlation analyses. This undermines confidence in subgroup-specific conclusions, especially when comparing them. Furthermore, the assertion that women are more affected than men may reflect differential measurement properties of QS-F/M, sociocultural reporting differences, or sampling variability, rather than biological or clinical disparities attributed to AD. In addition, as QS-F/M are sex-specific instruments with different constructs, comparing mean levels across sexes is not straightforward without standardization (e.g., by item-response theory transformation).

Beyond the burden imposed by AD, sexual function may be influenced by several factors, including relationship status, medication, menopause/andropause, obesity, comorbidities, affective disorders, and cultural and religious beliefs.^5^ Consequently, the correlations between disease severity and QS scores are likely influenced by these variables, hindering causal interpretation.

Thus, we encourage reporting internal consistency, item performance, and standardized QA-F/M scores for sex comparison. Also, clarifying whether QS-F/M analyses were restricted to sexually active participants would present a sensitivity analysis restricted to them.

Moreover, the inclusion of a broad age range (18–79 years) introduces heterogeneity, as age-related physiological and psychosocial changes can modulate sexual function. The lack of a matched control group (healthy individuals or other chronic dermatoses) substantially limits generalizability. Comparisons with population norms for EQ-5D-3 L or QS-F/M do not adequately substitute for a contemporaneous control group because they do not account for age, sex, socioeconomic status, or comorbid mental health conditions.

Therefore, the generalization of these findings and the inference that AD exerts a severity-dependent and sex-specific impact on sexual function should be interpreted with caution, given the observational design, potential confounding, and lack of an appropriate control group.

## Authors’ contributions

Vanessa Rigoni Marcato: Critical revision of the manuscript for important intellectual content; critical review of the literature; approval of the final version of the manuscript.

Anna Carolina Miola: Critical revision of the manuscript for important intellectual content; critical review of the literature; approval of the final version of the manuscript.

Paula Rosa Coutinho Goulart Borges Mariottoni: Critical revision of the manuscript for important intellectual content; critical review of the literature; approval of the final version of the manuscript.

Hélio Amante Miot: Conception and study design; data analysis and interpretation; manuscript drafting; critical revision of the manuscript for important intellectual content; critical review of the literature; approval of the final version of the manuscript.

## Declaration of generative artificial intelligence (AI)

During the preparation of this work, the authors used ChatGPT 5.2 to assist with English language editing. After using this tool, the authors reviewed and edited the content as needed and take full responsibility for the content of the published article.

## Financial support

CNPq (306358/2022-0) – Hélio Amante Miot is a CNPq researcher. CNPq (302789/2025-1) – Anna Carolina Miola is a CNPq researcher.

## Research data availability

Does not apply.

## Conflicts of interest

None declared.
